# Polypropylene-Based Porous Membranes: Influence of Polymer Composition, Extrusion Draw Ratio and Uniaxial Strain

**DOI:** 10.3390/polym10010033

**Published:** 2017-12-29

**Authors:** Pilar Castejón, Kian Habibi, Amir Saffar, Abdellah Ajji, Antonio B. Martínez, David Arencón

**Affiliations:** 1 Centre Català del Plàstic, Universitat Politècnica de Catalunya, C/Colom 114, Terrassa 08222, Spain; pilar.castejon@upc.edu (P.C.); kian.habibi@upc.edu (K.H.); antonio.martinez@upc.edu (A.B.M.); 2 Research Center for High Performance Polymers and Composite Systems, Chemical Engineering Department, Polytecnique Montréal, P.O. Box 6079, Montréal, QC H3C 3A7, Canada; amir.saffar@polymtl.ca (A.S.); abdellah.ajji@polymtl.ca (A.A.)

**Keywords:** polypropylene-based membranes, extrusion, porous morphology

## Abstract

Several commercial grades of homo-polymer and its blends were selected to prepare microporous membranes through melt extrusion-annealing-uniaxial stretching technique (MEAUS). Branched or very fluid polypropylene was employed to modify the polymeric composition. In some blends, micro-sized calcium carbonate was added. We analysed the influence of sample composition, extrusion draw ratio, and we performed a deep study concerning the uniaxial strain rate, using in some cases extreme strain rates and strain extents. The crystalline features were studied by Differential Scanning Calorimetry (DSC), and the morphology of porous structure was analyzed through Scanning Electron Microscopy (SEM). Thermal stability and thermomechanical performance was measured by thermogravimetric analysis (TGA) and dynamic-mechanical-thermal (DTMA) study. A close relationship was found between crystalline characteristics, porous morphology and the trends registered for permeability.

## 1. Introduction

Polypropylene (PP) membranes have been studied by several authors due to their wide use for commercial separation processes. Membranes can be formally categorized in different classes according to their chemical and physical characteristics and how they operate. The industrial membrane technologies can be classified based on the average pore diameter as follows: conventional filtration (10–100 µm), microfiltration (0.1–10 µm), ultrafiltration (1–100 nm) and reverse osmosis (~0.1 nm) [1]. Furthermore, polypropylene is a good candidate for certain industrial applications such as gas separation, Li-ion batteries or medical applications, due to its outstanding properties such as high melting temperature, chemical resistance and good mechanical properties [2,3]. 

Polymeric membranes are conventionally produced using the phase inversion process, in which solvents are used to form the pore network. To avoid the use of solvents, due to their cost, environmental footprint and contamination risks, a dry process based on the stretching of extruded film with a specific structure called MEAUS (melt extrusion-annealing-uniaxial stretching) was developed in recent decades. This process is applicable to semi-crystalline polymers, and three consecutive stages are carried out: (a) fabrication of a precursor film with a row-nucleated lamellar structure; (b) annealing the film to increase the lamellar thickness and removes defects in the crystalline phase; (c) stretching the annealed film at low and high temperatures to create and enlarge the pores [4,5,6,7,8,9,10,11,12,13,14,15,16,17,18,19,20,21,22,23,24,25,26]. After the stretching steps, the film should be heat-set in order to maintain dimensional stability and avoid shrinkage [6,24].

For the first stage, several studies have reported on the effects of processing parameters such as the extrusion conditions and the properties of the polymer or blends used [4,5,6,7,8,9,10,26,27]. The molecular weight, molecular weight distribution and polymer architecture are key material parameters in order to create a suitable lamellar structure. Sadeghi et al. [7] selected different grades of PP to investigate the influence of molecular weight on the lamellar structure. They concluded that molecular weight is a critical factor in the control of orientation and uniformity in the crystalline phase, and consequently of the pore interconnectivity. In another study, Sadeghi et al. [8] showed that adding a small amount of branched polypropylene increases the number of entanglements during melt stretching and creates a specific structure containing long elongated fibrils with thinner lamellae. This structure improves the membrane permeability due to the formation of larger amount of pores.

The other processing parameters, such as draw ratio and air cooling conditions were examined by Tabatabaei et al. [9]. They reported that increasing the draw ratio as well as applying air cooling right at the exit of the die, have a strong effect on the crystalline orientation. In order to examine the effect of orientation on the performance of the films, tensile characterization was carried out by Sadhegi et al. [10]. The results showed two distinct regions in the stress-strain curves for the annealed samples. They attributed the first elastic region to the stretching of the short tie chains, while the second region was due to the void creation because of chain scissions, and the bridges formation after the crystallization of longer chains located between crystal blocks.

Many works have been carried out in order to analyze the effect of the annealing procedure on the morphology of the resulting membranes. This is a critical stage where the lamellar thickness and regularity are improved. Some authors investigated the influence of applying different annealing times and temperatures for polypropylene precursor films, and concluded that a minimum temperature of 135 °C and time of 480 s was necessary to form appropriate secondary crystalline lamellae between the primary row-nucleated structure [16,17,18,19,20,21]. Subsequently, during cold and hot stretching stages these microsecond crystals convert to connecting bridges, which resulted in the development of the pore structure [11,22,23]. Saffar et al. [11] investigated the influence of stretching variables on the morphology and permeability of microporous membranes. They found that larger porosity and permeability values were obtained by stretching at a low strain rate, and at a stretch ratio for the cold stretching step below an optimum percentage in order to avoid a small number of non-uniform pores, as well as when the hot stretching temperature and stretch ratio was increased. 

Several studies have focused mainly on the use of neat polyolefins. However, only a few works have dealt with filled polyolefin. Nakamura [12] and Nagō [13] created pores through the debonding of CaCO_3_ from the polymeric matrix by biaxially stretching, and evaluated the dependency of the draft ratio in relation to orientation and properties of the microporous polypropylene sheets. The use of mineral fillers can provide to the membrane an increase in rigidness and an enhancement of the hydrophilic surface characteristic. It can also affect the final porous morphology of the membranes, as most of the fillers have some nucleate ability in the crystallization processes of polyolefins. In this sense, some works claim that this nucleating effect of calcium carbonate [28,29] can have an effect on the crystalline microstructure generated.

In the present work, we evaluate the influence of the polymer matrix composition and the architecture structure on the row-nucleated lamellar crystallization by using two linear polypropylenes having different molecular weights, and blending with branched and very fluid PP resins. Some researchers have investigated the fabrication of microporous membranes from blends of polypropylenes with different molecular weights and different low molecular weight tail contents [4,26]. For this investigation, we have used an extremely fluid polypropylene (adding up to 10%) in order to try to achieve a greater strain extent during the uniaxial strain stage, with the purpose of enlarging the pore size as much as possible. As a complement, we have performed a detailed investigation of the impact of all the variables on the uniaxial strain stage (stretching both at room and high temperatures), related to the structural changes produced in the microporous morphology. In some cases we have used extreme values of strain rate and larger strain extents than what is usually applied (up to 300%). The resulting permeability is based on the close relationship observed between crystalline features (through FTIR and Differential Scanning Calorimetry (DSC)) and porous morphology. Micro-sized ultrafine surface-treated precipitated calcium carbonate filler has never been used before for the manufacture of membranes using the MEAUS technique. We hypothesized that this well-known filler, employed in the industry for its effect on the polypropylene matrix, would lead to changes in the crystalline structure, which would, in turn, affect to the row-lamellar structure. This effect, along with debonding mechanisms during the strain stage, could have a synergistic effect in the generation of bigger pores. Furthermore, the membrane isobutene permeability was also determined, in the interest of assessing the usefulness of membranes obtained by MEAUS technology as an integral component in lighters.

## 2. Materials and Methods

### 2.1. Sample Compounding

Four commercial grades of homopolymer polypropylene (Table 1) were kindly supplied by Repsol (Madrid, Spain), renamed in this paper as H1, H2 and H-VF (very fluid) and Borealis (Vien, Austria), renamed in this paper as H-BR (branched). They had different molecular weight distributions and chemical architectures, and their main characteristics are shown in Table 1. Micro-sized (4μm average size) ultrafine surface-treated precipitated calcium carbonate (Reverté Minerals, Barcelona, Spain) was added as filler.

With these raw materials, several polypropylene based compounds were prepared through melt extrusion blending. A twin-screw extruder Collin ZK-35 (Dr Collin GmbH, Ebersberg, Germany) was used with a temperature profile from hopper to die of 140–210 °C, and a screw speed of 105 rpm, and a circular die of diameter 3 mm. The extrudate was cooled in a water bath and pelletized. Pure polymers were also extruded at these conditions in order to provide the same thermomechanical history to all the samples. Finally, seven samples were obtained: unfilled samples such as H1 and H2 (100 wt % polypropylenes), H1-2BR (98–2 wt %), H1-2VF (98–2 wt %), H1-10VF (90–10 wt %), and calcium carbonate filled samples named H1-C5 and H1-C10, containing 5 and 10 wt %, respectively, of calcium carbonate.

### 2.2. Extrusion and Annealing of Precursor Films

**Figure 1:**
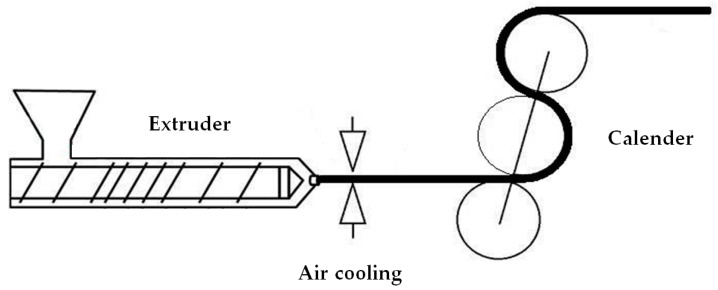
Summary outline of the fabrication of the precursor films.

The first stage of the MEAUS process is the extrusion of mono-oriented films with a high draw ratio and a rapid cooling of the extrudate at the exit of the extrusion die while drawing (Figure 1). The product of this process is normally called precursor film, as its structure would allow to the generation of a row-lamellar structure that could be transformed into a porous membrane.

A single screw extruder (Eurotecno, Sant Fost de Campcentelles, Spain), L/D = 35, equipped with a slit die of 200 mm width and variable thickness was used. The temperature profile from hopper to die was 140–230 °C. At the exit of the die, slit-open air knives were used to supply air at a pressure of 5 psi to both sides of the film in order to achieve fast cooling. At the same time, the film underwent uniaxial stretching along the machine direction using a calendar system with variable speed, which allowed the application of different draw ratios to the extruded precursor films. The draw ratio was determined by dividing the linear speed of the molten material at the exit of the die by the linear calendaring rolling speed. All the precursor films were then annealed at 140 °C for 15 min in order to eliminate crystalline imperfections and to thicken the crystalline lamellae.

### 2.3. Uniaxial Strain of Annealed Precursor Films

The annealed precursor films were uniaxially stretched in two stages. The first stage was carried out at room temperature (cold stage), whereas the second stage was performed at 140 °C (hot stage). For uniaxial strain, a universal testing machine equipped with a climatic chamber was employed. Rectangular samples with dimensions of 100 mm × 60 mm were cut out from the annealed precursor films and were tighten in two grips in tensile configuration. The direction of the uniaxial strain was parallel to the direction of the drawing of the precursor film during the extrusion. For both the cold and hot stages, two parameters were subjected to study: the crosshead speed and the percentage of strain reached (from now on referred to as strain extent). Once the test was finished, before relieving the films from the imposed strain, all of them were kept at 140 °C for 90 s in order to stabilize the porous structure.

### 2.4. Membrane Characterization

Fourier-Transmission Infrared spectra were carried out using a Perkin Elmer Spectrum 1000 FTIR (Perkin Elmer, Waltham, MA, USA) (resolution 1 cm^−1^) and polarized beam. The crystalline orientation was determined based on the difference of the absorption in the two orthogonal directions, parallel (*A*_0_) and perpendicular (*A*_90_) to the reference axis (extrusion machine direction). The crystalline phase orientation (*F*_c_) of polypropylene was obtained from the absorption at a wavelength of 998 cm^−1^. The Herman orientation function was calculated according to Equation (1), where *D*, dichroic ratio, is *A*_0_/*A*_90_.

*F*_c_ = (*D* − 1)/(*D* + 2) (1)

Differential Scanning Calorimetry with a DSC Q2000 (TA instruments, New Castle, DE, USA) aimed to analyze changes in the crystalline distribution in the porous materials produced. Calibration of the instrument was done using standard samples of indium and lead. The sample mass was typically 5–7 mg. Heating runs between 40–200 °C were performed at a heating rate of 10 °C/min. The crystallinity percentage of the polypropylene was calculated according to Equation (2), where Δ*H*_m_ was the melting enthalpy measured in heating experiments. Δ*H*_0_ is the theoretical enthalpy of PP 100% crystalline (207.1 J/g), m_c_ the mass of the sample, and *m*_p_ the mass of PP in the sample.

*X*_m_ = (Δ*H*_m_∙*m*_c_∙100)/(Δ*H*_0_∙*m*_p_) (2)

A TGA/DSC 1 Mettler Toledo Star System (Mettler Toledo, Columbus, OH, USA) analysis was used to evaluate the thermal stability of the membranes with different compositions by performing thermogravimetric analysis. This was achieved by heating samples of around 8.0 mg from 40 to 700 °C at 10 °C/min. A constant running flow of air (60 mL/min) was used in the experiments. Three different measurements were done for each membrane.

Dynamic mechanical thermal analysis (DMTA) was performed on membranes with different compositions. Specimens were tested in a DMAQ800 machine (TA Instruments, New Castle, DE, USA) operating in a tensile testing mode. A gauge length of 14 mm was used. The test specimen was cooled to below −40 °C, allowed to stabilize and then was heated at a rate of 2 °C/min to 140 °C. A static stress of 1 MPa was applied to ensure that the membrane was taut between the tensile grips, and a sinusoidal (dynamic) tensile load was applied to produce a dynamic strain with constant amplitude of 0.2%.

The morphology of the generated porous structure was checked via scanning electron microscopy (SEM) with a JEOL JSM-5610 microscope (7kV voltage) (JEOL, Akishima, Japan). The samples were coated with a thin layer of gold alloy in an argon atmosphere using a BAL-TEC SCD005 Sputter Coater (BAL-TEC, Balzers, Liechtenstein). The number of pores divided by the analyzed membrane area provided the average pore density. The percentage of porous surface area of the membranes was obtained using an image analysis program (Omnimet Advanced, Buehler, Lake Bluff, IL, USA, 2015). For calculation purposes, a circular-like porous geometry was assumed.

A value of porosity was estimated by imbibition of the samples into the water. Then we used the grain volume method to determine the porosity of samples. Therefore, the first *V*_p_ of samples was computed by Equation (3):

*V*_p_ = (*W*_sat_ − *W*_dry_)/ρ_water_ (3)

where saturated weight is (*W*_sat_), dry weight of sample is (*W*_dry_) and density of a certain fluid which is water in this study is ρ_water_. Hence, Equation (4) gave *V*_g_, where σ_objective_ is the density of the samples.

*V*_g_ = *W*_dry_/ρ_objective_ (4)

Then the percentage of porosity (Φ) was obtained by dividing *V*_p_ by *V*_b_ as given, where *V*_b_ is the sum of *V*_p_ and *V*_g_:

Φ = (*V*_p_/*V*_b_) * 100 (5)

The permeability to air of the porous materials was characterized through a Gurley Densometer Lorentzen & Wettre (ABB, Zurich, Switzerland), according to ISO 5636-5. The time (*t*) for a settled volume (100 mL) of air to pass through the sample with a fixed area (0.79 cm^2^) under the pressure of 0.02 MPa was measured. Generally, longer times correspond to low air permeability and a long tortuous path for air transportation, implying higher curvature of pores. A value of Gurley permeability was obtained through Equation (6):

Gurley permeability = 135.5/tμm·Pa^−1^·s^−1^ (6)

Testing for permeability to isobutene was carried out in several zones of the obtained membranes during the analysis of the uniaxial strain conditions. Measurement area was around 7 mm^2^. The isobutene flow rate was determined using the flowmeter FESTO SFET-R0010-L-WQ4-DK3 (FESTO, Esslingem am Nacker, Germany), with a measurement range of 0 to 100 mL/min.

## 3. Results

The exposition of the obtained results will be divided according to three main headings: composition of the samples, draw ratio applied in the production of precursor films, and the influence of the variation of crosshead speed and strain extent during the uniaxial strain stage.

### 3.1. Influence of Polymer Composition

All the membranes discussed in this section were obtained under the same processing conditions. The extrusion draw ratio was kept to 80, (giving as a result a precursor film of about 30 μm) and the uniaxial strain stage was carried out during the cold stage at 10 mm/min, up to 55% strain extent, and during the hot stage at 10 mm/min, up to 300% strain extent.

**Figure 2:**
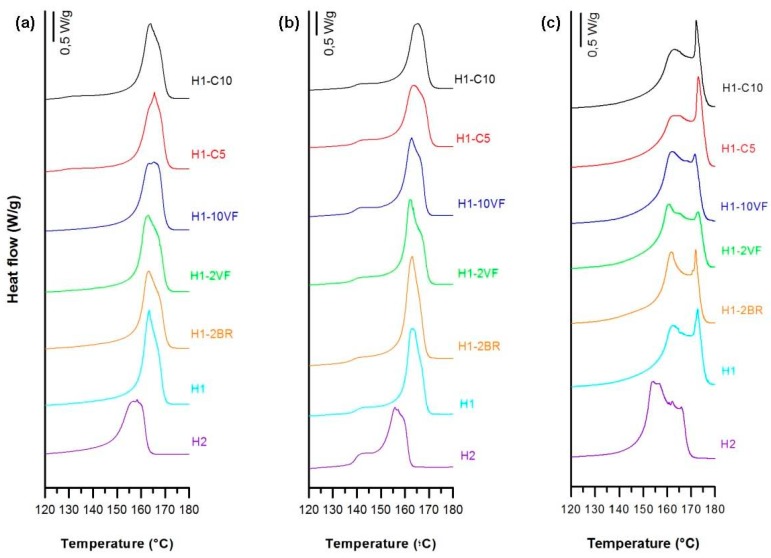
Thermograms differing in sample composition, (**a**) precursor films; (**b**) annealed precursor films; (**c**) membranes.

All numerical results of this section are summarized in Table 2. As depicted in Figure 2a,b, the significant changes among the melting endotherm curves for the neat resins and the blends are shown by the presence of two distinctive shoulders on both sides of the melting peak. For the homopolymer resins, the main melting peak in these thermograms is attributed to the presence of thinner and/or thicker lamellae distribution of the original crystalline structure obtained in the precursor films [7]. The shoulder at lower temperature is due to the annealing process, when the mobility of the polymer chains in the amorphous region increased due to the higher annealing temperature. During this step, chains located in the amorphous part would have rearrange themselves and recrystallize. Then, the crystalline growth would have occurred around the initial row-nucleated lamellae, creating new secondary crystals, as has been reported by other authors [14,16,17,18,19,20,21]. For the annealed precursor films, the melting peaks were quite similar except for the sample H2, where the lower molecular weight led to changes in the crystalline structure (lamellae thickness and orientation). Blends with the very fluid polypropylene (H1-2VF and H1-10VF) had reduced total crystallinity. High molecular weight resins that possessed the long macromolecules with larger relaxation time and the longer molecular chains kept the promoted orientation under fast cooling. The blend with the branched polypropylene (H1-2BR) also had a reduction in crystallinity and orientation of the crystalline phase. This phenomenon could be explained through the lens of growing lamellar blocks that can twist and thus decrease the overall orientation [10]. Calcium carbonate filled samples showed a low-temperature endotherm peak around 130 °C in the non-annealed precursor films when compared to unfilled samples. This is in concordance with the results obtained by Supaphol et al. [28], and it is related to a double peak during the crystallization. For the annealed precursor films, this secondary endothermic plateau moved to higher temperatures and it was more intense, similar to unfilled samples, due to the improvement of the crystalline structure during the annealing process.

**Figure 3:**
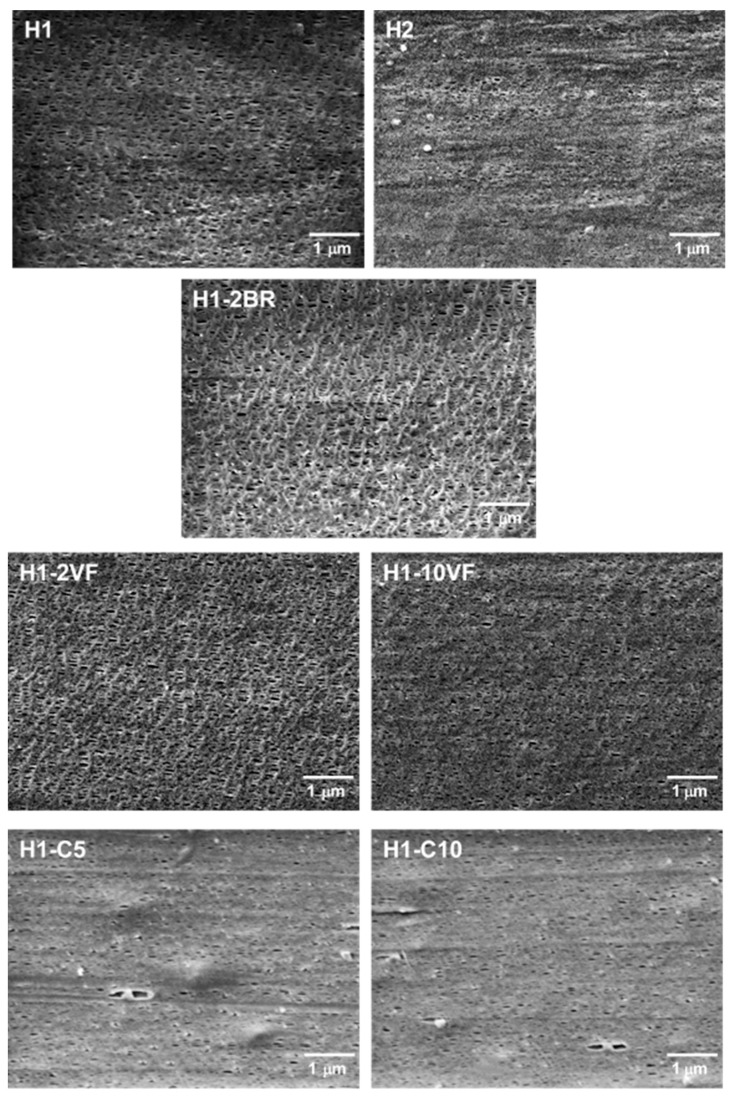
Scanning Electron Microscopy (SEM) micrographs differing in sample composition.

Thermograms of membranes (Figure 2c), showed a shoulder at higher temperatures related to the lamellae redistribution after stretching. During the cold and hot stretching step, pores were created and enlarged due to the stretching of short and long tie chains. This resulted in a local crystallization, which explains the appearance of interconnected bridges between lamellae. The presence of this new and stable recrystallized structure also caused the disappearance of the left plateau related to the annealed precursor films [7,11,20,21,22,23]. This right shoulder is more pronounced in samples H1 and H1-2BR, which is evidence of the generation of a larger number of interconnected bridges. Pore morphology micrographs (Figure 3) seem to be related to the increase in interconnected bridges, as the pores in H1 and H1-2BR were larger in size and density. The addition of very low molecular weight polypropylene (VF) caused a gradual reduction in pore density, as well as in porous area. It seems that short chains affect the relaxation time of macromolecules during the extrusion process and, consequently, there was a drop in the number of nucleating sites. It is also obvious that a more intense lamellar structure was obtained when branched polypropylene was added, due to the longer relaxation time of macromolecules. Calcium carbonate shifted this secondary shoulder to higher temperatures. In this case, the effect of the double peak during crystallization could also affect the main endothermic signal.

For both filled and unfilled samples, there was a close correlation between the percentages of porous area and the Gurley permeability values. This accounts for the basic mechanism of this kind of membranes, acting as a flux restrictor, depending on the pore size and effective porous area. The synergistic effect of the MEAUS process in filled samples was noticeable due to the combination of the generation of pores because of the separation of lamellar blocks, along with the debonding mechanisms of calcium carbonate from the polymeric matrix (Figure 3) which created larger pores than the ones obtained only by MEAUS, leading to an increase of permeability.

**Figure 4:**
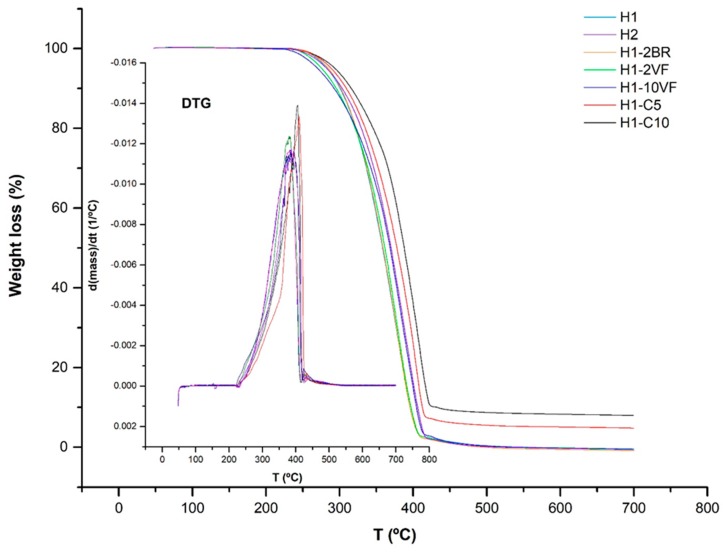
Plots of thermogravimetric analysis (TGA) of membranes with different compositions.

The thermogravimetric analysis (TGA) and its respective first derivative (DTG) for all the membranes studied are shown in Figure 4. From these plots, values were collected for the temperatures at which there was a loss of mass of 10 wt % (T_0.1_) and 50 wt % (T_0.5_), the relative lost mass at 400 and 600 °C, and the temperature of maximum lost mass velocity (T_max_) (Table 3). All TGA curves correspond to one-single step process that can be described through an apparent first order mechanism, where the only variable is the mass conversion into volatiles [30]. This feature can also be seen in the DTG curves, where one single peak is detected.

Some data in the literature [31], have evidenced the influence of air traces adsorbed in the amorphous phase of the initial degradation states of pyrolysis. The results of crystallinity obtained by DSC (Table 1) allow us to reasonably conclude that compositions having low molecular weight polypropylene can retain less oxygen content due to the reduced amount of amorphous phase. The improvement in the thermal stability of membranes containing calcium carbonate is because of the formation of char residues (at 600 °C), which hinders the out-diffusion of the volatile decomposition products. Mineral fillers have higher volumetric heat capacities and thermal conductivities than polypropylene [32], thus the filled membranes would absorb more heat as compared to the pure polypropylene membrane. Because of the colligative thermodynamic effect, the temperature of the filled membrane would increase and the polypropylene would start to degrade at higher temperatures.

**Figure 5:**
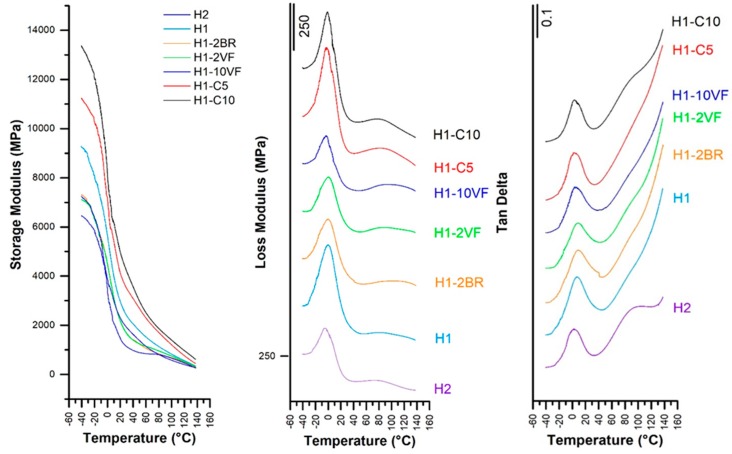
Storage modulus, loss modulus and tan δ evolution.

Concerning the DMTA analysis (Figure 5), several facts can be extracted. First, the molecular weight of polypropylene in H1 (highest molecular weight) had the effect of increasing the storage modulus values (E’) at all temperatures (Table 3). The use of high proportions of very fluid polypropylene (H1-10VF) significantly reduced the storage modulus values. This trend is not the same as the one observed by Arranz-Andrés et al. [33] for metallocenic grades of homopolypropylene with different molecular weights. In their work, they used solid samples obtained by compression molding, but in our work, our samples were porous membrane obtained with a process that involved high orientation. That means that the orientation magnified the incidence of the molecular weight of polypropylene. In addition, it must be taken into account that the tests were performed under tensile configuration, along the orientation induced during extrusion, so this effect could be even be more magnified. For filled membranes, the presence of a rigid phase increased the storage modulus, being as these samples were the most rigid of all the studied membranes.

The glass transition, (β-relaxation) was also affected by molecular weight. More differences that are significant were observed when comparing the loss modulus peaks with tan δ values. It was observed that the addition of 10% very fluid polypropylene increased the general mobility of polypropylene chains, and that the lower molecular weight of H2 with respect to H1 decreased these values. Quite surprising was the lowering effect of calcium carbonate on T_g_. Kamal et al. [34] reported that small calcium carbonate particles displaced this β-relaxation peak to lower temperatures, and that this was related to the surface treatment. In our case, the calcium carbonate was surface-treated with a mixture of MgCO_3_, Fe_2_O_3_ and amino groups.

### 3.2. Influence of Draw Ratio

**Figure 6:**
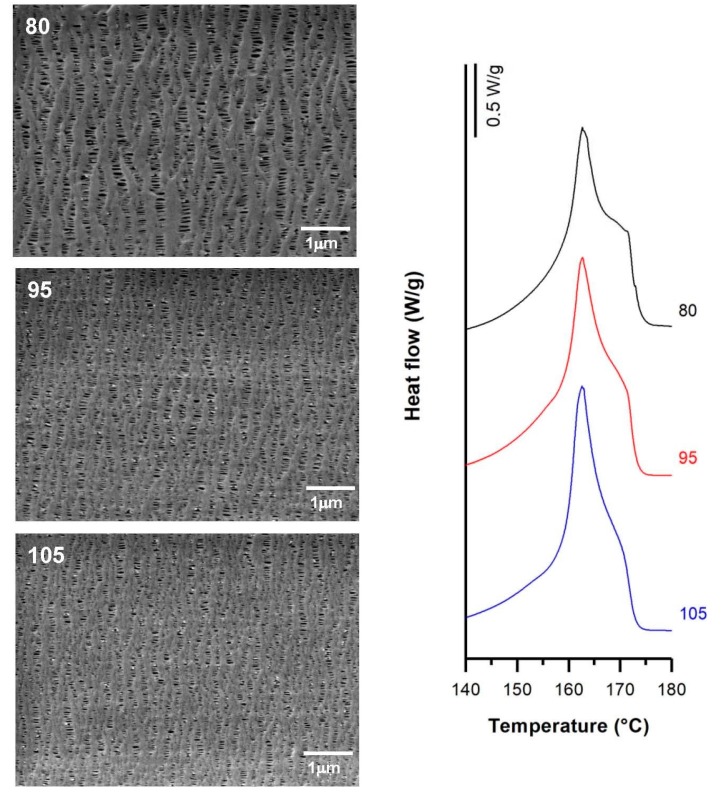
SEM micrographs and Differential Scanning Calorimetry (DSC) thermograms of H1-2BR, differing in their draw ratio. Processing conditions: Gap die, 1.9 mm; cold stage 0.3 mm/min, 20%; hot stage, 0.3 mm/min, 130%.

For this study, H1-2BR and H1-C10 samples were selected, and the numerical results obtained are presented in Table 4. For non-filled samples, the membranes obtained from precursor films with higher draw ratios displayed a more pronounced shoulder in DSC thermograms (Figure 6) related to the formation of a larger number of interconnected bridges. The higher crystalline orientation in the precursor films resulted in the generation of a larger number of pores. These results are in agreement with the findings of other authors [7,9]. Before the annealing process, where the properties of the crystalline structure necessary to obtain the microporous structure are improved, it was necessary to increase the draw ratio applied during the extrusion step. Under these conditions, an optimal row-nucleated structure was achieved when the lamellae aligned themselves perpendicular to the machine direction. This enhanced the crystal thickness as well as the orientation of the crystalline and amorphous phases. Conversely, a higher orientation value led to the reduction of the amorphous tie chains mobility [15], limiting the pore enlargement (Figure 6). No significant effects were observed in Gurley permeability values, as they remained practically constant.

**Figure 7:**
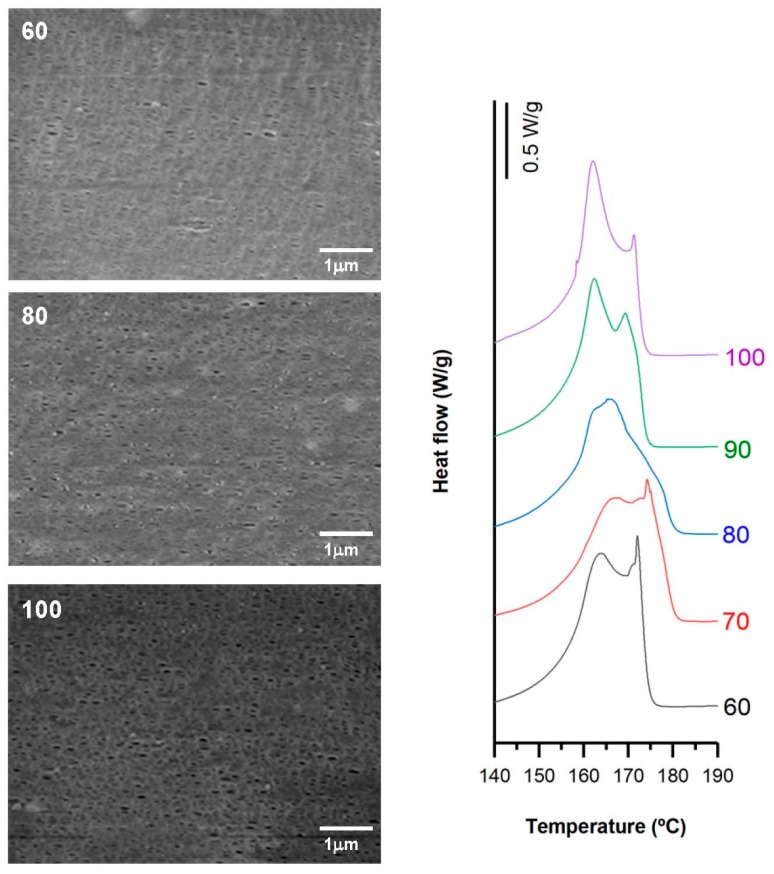
SEM micrographs and thermograms of H1-C10, differing in their draw ratio. Processing conditions: Gap die, 1.9 mm; cold stage 0.3 mm/min, 20%; hot stage 0.3 mm/min, 130%.

Filled samples (H1-C10) also showed a noticeable increase in the orientation of the crystalline phase when high draw ratios were imposed during the extrusion process. As the draw ratio increased, a change in the bimodal distribution of the melting peak was noticed in the thermograms (Figure 7). Lower draw ratios led to a more intense second peak, whereas higher draw ratios inverted this trend. In this sense, the highest draw ratio provided finer precursor films that cooled more rapidly, and thus less-than-perfect crystalline entities were formed during the extrusion stage. As a result, the final membrane showed bimodal distribution, but with a reduction in the population of more perfect crystalline entities. A maximum pore density and porous area was reflected by the highest Gurley permeability value registered. The limitation of pore enlargement observed in non-filled samples was attenuated in filled samples, probably due to the contribution of debonding mechanisms of the filler from the polymeric matrix. 

### 3.3. Influence of Uniaxial Strain Experimental Parameters

A deep analysis of the experimental conditions that affect the uniaxial strain stage of the global process is presented in this section. All the samples were obtained by using a gap die of 1.9 mm and a draw ratio of 90in the extrusion stage. The material employed in all cases was H1. Variations of strain rate and extent of deformation were performed in the cold and hot stages. Table 5, summarizes the conditions applied and the results in terms of crystallinity, pore morphology and permeability to air. All figures in this section show the close relationship between pore morphology (SEM micrographs) and crystalline entities (DSC thermograms).

#### 3.3.1. Effect of Strain Rate

Applying larger strain rates during lamellae separation raised the number of pores. This is attributed to breakage in a greater number of tie chains due to less flexibility of the amorphous phase at room temperature. The reduction in the molecular motion with increasing strain rate also limits the separation of lamellar blocks, and thus the pore enlargement. In membranes prepared at higher strain rates during the cold stage, the contribution of shoulder peaks attributed to connecting bridges was lower. This may be due to the lower presence of recrystallized bridges when the strain rate was increased and some pores collapsed [17]. However, membranes prepared at lower strain rates at room temperature showed slightly larger porosity and Gurley permeability values. This made it possible to obtain membranes with similar properties with a significant reduction in testing time.

**Figure 8:**
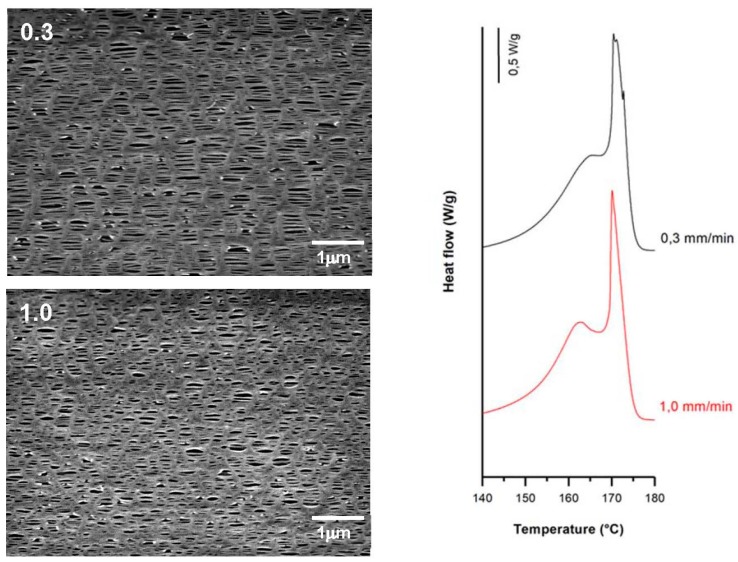
Influence of same cold and hot strain rates (upper-left, in mm/min). Common uniaxial strain conditions. Cold stage= 35%; hot stage = 320%.

**Figure 9:**
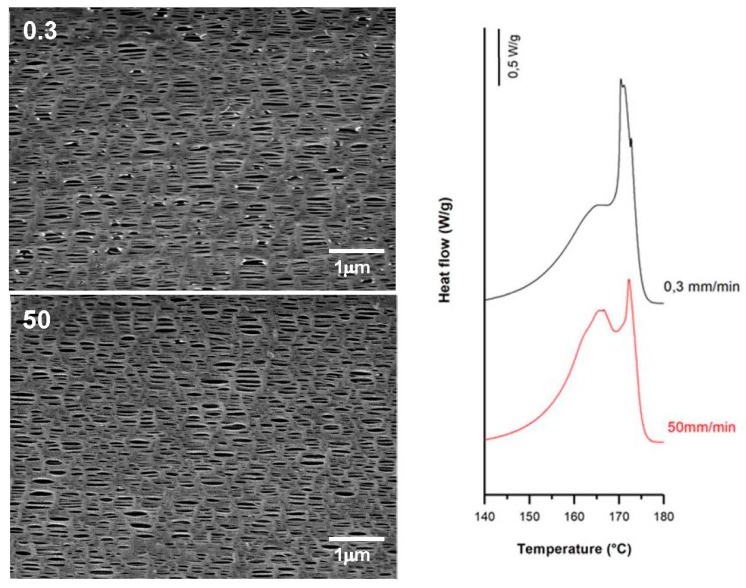
Influence of cold strain rate (upper-left, in mm/min). Common uniaxial strain conditions. Cold stage, 35%; hot stage, 0.3 mm/min, 320%.

**Figure 10:**
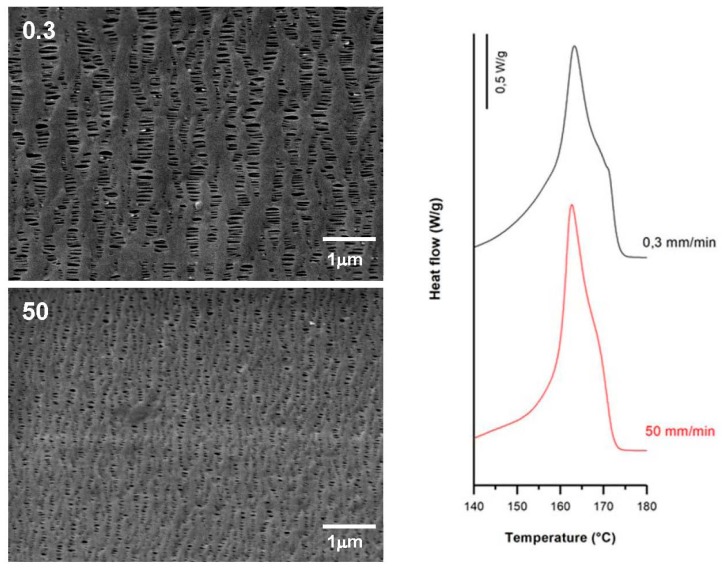
Influence of hot strain rate (upper-left, in mm/min). Common uniaxial strain conditions. Cold stage, 0.3 mm/min, 20%; hot stage, 130%.

According to the theory proposed by other authors [7,8,9,10,11,15,16,17,18,19,20,21,22,23,24,25], pores that were created during the cold extension were enlarged during the hot stretching step as the stress increased linearly. This is due to the larger flexibility of the tie chains that formed the interconnected bridges between the lamellae structures. SEM micrographs and DSC thermograms (Figure 8, Figure 9 and Figure 10) showed a reduction in pore size, reduced lamellar separation and shorter interconnecting bridges when higher strain rates were employed. This was due to the low chain mobility and strong crystalline structure. Higher porosity and Gurley permeability values were obtained when applying lower strain rates at higher temperatures as a result of the lower resistance exerted by lamellae separation [11]. Lower pore density and larger pore size for membranes stretched at lower rates could be explained as a possible collapse of pores due to breakage in some connecting bridges. In this stage, some chains could be pulled out from the initial lamellae and recrystallized, forming a thicker and more uniform crystalline structure [20,21,22,23].

#### 3.3.2. Effect of Strain Extent

**Figure 11:**
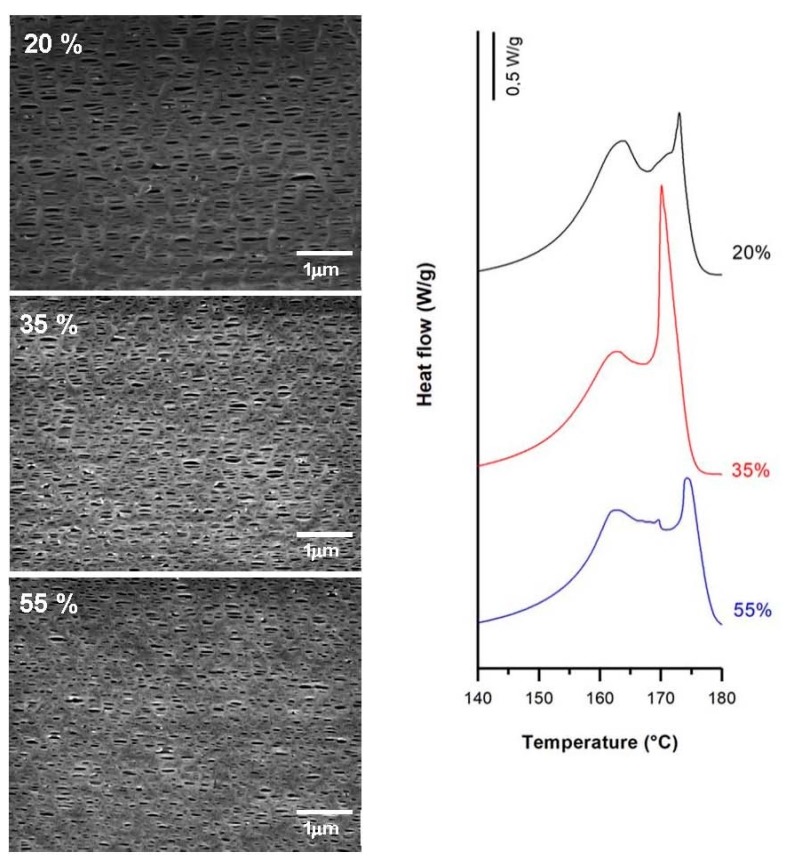
Influence of cold strain extent. Common uniaxial strain conditions. Cold stage, 1.0 mm/min; hot stage, 1.0 mm/min, 320%.

Secondary crystals formed during the annealing process and some chains from the initial lamellae structure created the connecting bridges during cold and hot stretching [20,21,22,23]. The low mobility of macromolecules and the increase of strain extent during the cold stage also led to the formation of new pores due to chain scission during lamellae separation. Conversely, the effect of applying a higher percentage beyond an optimum value of about 35% for the cold stretching step caused a reduction in pore density (Figure 11). An explanation could be the lamellae fragmentation produced at higher stretch values, which may have generated a reduction in the crystalline orientation and a collapse of pores when fibrils got closer to each other due to an elastic response [4,11]. It is of note that this optimal value in cold strain extent is related to a very intense secondary melting point (contribution from connecting bridges) in the main membrane melting peak (Figure 11).

**Figure 12:**
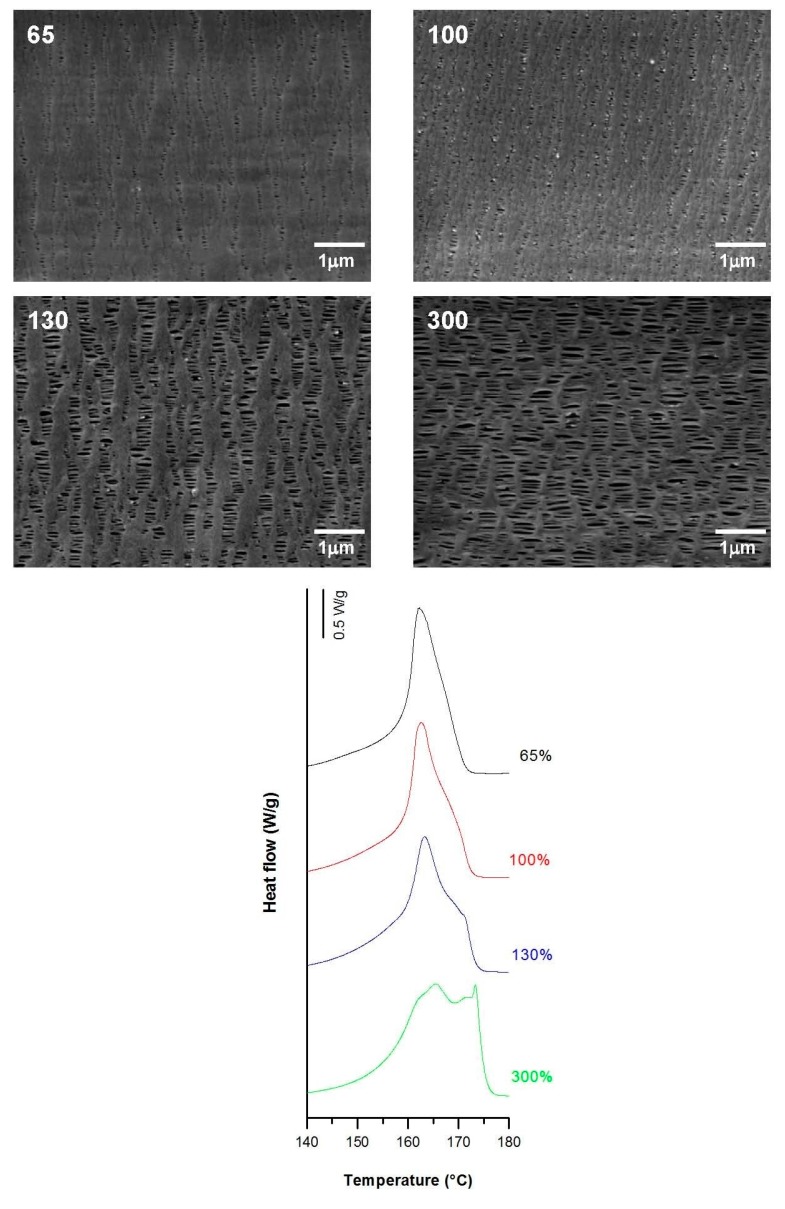
Influence of hot strain extent. Common uniaxial strain conditions. Cold stage 0.3 mm/min, 20%; hot stage, 0.3 mm/min.

The influence of applying a high stretch ratio (up to 300%) during the stretching step at high temperatures was investigated. The larger the hot stage extension, the greater the pore size and uniformity. This phenomenon could be attributed to the higher flexibility and mobility of the macromolecules at elevated temperatures, resulting in a greater lamellae separation, as well as the enlargement of some invisible pores after cold extension. The sample stretched to 300% had a smaller number of pores but larger average pore size compared to previous samples (Figure 12). This could be the result of the breakage of some connecting bridges that reduced its thickness with a higher stretch ratio. Porosity and thus Gurley permeability was extremely dependent on the global strain extent, and their values increased with increases in the global strain. A noticeable growth in the pore size was evident in conjunction with the gradual apparition of a secondary shoulder in the main DSC peak at higher strain extents. This also related to the presence of longer recrystallized connecting bridges (Figure 12).

#### 3.3.3. Practical Case: Membranes as Integral Parts of Lighters

**Figure 13:**
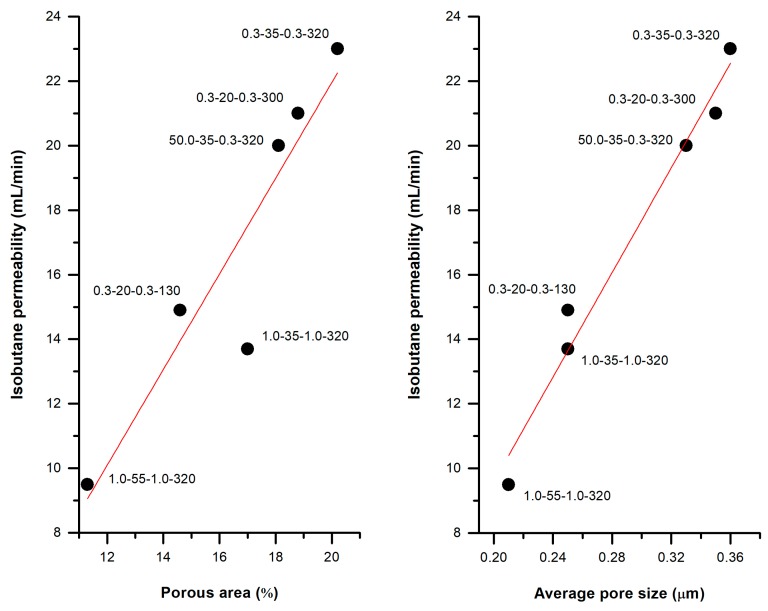
Isobutane permeability of some H1 membranes. Each point refers to the following sequence: crosshead speed (cold), strain extent (cold), crosshead speed (hot), strain extent (hot).

These membranes can be used as integral parts of lighters, regulating the flux of gas that is necessary to create a flame. Most lighters use isobutylene as the substance responsible for creating the flame. Figure 13 shows the linear trend of the permeability of some of the membranes studied in the uniaxial strain analysis with respect to the porous morphology developed. This linear trend is a confirmation of the permeation mechanism of this kind of porous materials, acting as a flux restrictor, depending on the pore size and the porous area.

## 4. Discussion and Main Conclusions

In this work, we investigated the influence of polymer composition on the structure of filled and non-filled PP membranes. The results support the idea discussed in previous works [7,8,9,10] regarding the high importance of the polymeric matrix nature in the generation of precursor films which are capable of producing a row-lamellar crystalline structure. Based on the SEM micrographs and DSC results of filled and non-filled neat polypropylenes with different molecular weights, and blends with branched and very fluid PP resins, the discussion of our results can be summarized as follows.

Lower porosity and permeability values were observed when we employed low molecular weight polypropylenes, such as H2 and, above all, VF. The use of these polymers is counter-productive with regards to keeping a macromolecular alignment for long enough duration for the generation of a row-lamellar crystalline structure. The hypothesized positive effects with regards to greater deformation during the uniaxial strain stage were restricted by the macromolecular rearranging. In addition, it has been observed that increasing the strain extent during the uniaxial strain stage increased the pore size, due above all to the coalescence of close neighbor pores. In this case, these bigger pores tend to have an elliptical shape. This has a consequence in terms of the Gurley permeability values registered: passing from a total strain extent of 130% to 300% (Table 5) did not substantially increase the permeability of the membrane. On this topic, the authors are currently working on enlarging pore size without losing the spherical shape of the pores. To this end, they are using heterophasic copolymers of polypropylene-ethylene in order to enhance the quality of the deformability of the polymeric matrix without losing macromolecular orientation during high draw ratio extrusion processing.

The combination of MEAUS technology in filled polymers opens a wide range of porous and crystalline morphologies, as well as enlarging the range of permeability values. In this work we have noted that the addition of calcium carbonate helped to increase the population of more perfect crystalline entities, with a more intense secondary melting peak observed (Figure 2) at higher temperatures. Above all, the highest permeability observed for filled membranes when compared to unfilled one must be attributed to the debonding of filler from polymer during the uniaxial strain stage, as observed in SEM micrographies (Figure 3), where there were some areas in which a remarkable void was generated near a calcium carbonate particle. Due to the potential of using MEAUS processing for mineral filled compounds, the authors are currently working in mineral filled polymeric systems, employing wide-range industrial mineral fillers, such as talc.

Calcium carbonate raised the thermal stability and rigidity of the membranes. It was also noted that the use of low molecular weight polypropylenes helped to enhance the thermal stability of the membranes, and affected the glass transition temperature.

With regards to aspects related only to the extrusion processing parameters, there was a higher dependence on the crystalline phase orientation than on the extrusion draw ratio [9,10]. This imposed macromolecular alignment along the extrusion direction is very relevant concerning the final morphology of the membranes. By increasing the applied draw ratio during the extrusion step, a macromolecular alignment along extrusion direction was imposed, and optimal row-nucleated structure concerning the final microporous membrane morphology was achieved. For the filled membranes with CaCO_3_, the higher the draw ratio applied, the higher the porosity and Gurley permeability values obtained due to the contribution of debonding mechanisms of the filler in the polymeric matrix. Nevertheless, for non-filled samples, values above a certain draw ratio did not show this same response, since the higher crystalline orientation limited the pore enlargement and no higher porosity or permeability values were achieved.

Likewise, the uniaxial strain stage was decisive in the porous morphology of membranes. Using extreme strain conditions, that is, very low strain rates or very high strain extents, it was possible to generate a wide range of porous morphologies which were directly related to the permeability performance. Main results of this detailed study can be summarized as follows:

Slight porosity and Gurley permeability were obtained by applying very low strain rates for stretching at room temperature. However, related to the strain ratio applied during this cold stretching step, there was an optimum extent percentage close to 35%, which produced membranes with higher porosities. 

During stretching at high temperatures, when the mobility of macromolecules was increased, a slower strain rate and a higher strain extent had a significant effect on the final porous morphology. Changes in the crystallized structure during this step were produced and higher permeability values could be obtained mainly due to pore enlargement with greater lamellae separation, until a total collapse of the crystalline structure occurred. Nevertheless, it should be taken into account that these extreme conditions would lead to longer production times. A strong linear relationship between the porous area and the permeability to isobutene has been found.

## Figures and Tables

**Table 1:** Commercial grades of polypropylene employed.

Commercial Tradename	Code	Polymer Architecture	MFI ^a^ (dg/min)
ISPLEN PP020 G3E	H1	Linear	0.9
ISPLEN PP034 W3F	H2	Linear	2.2
ISPLEN PP099 K2M	VF	Linear	55.0
DAPLOY WB140HMS	BR	Branched	2.1

^a^ MFI (Melt Flow Index) measured according ISO 1133 (230 °C, 2.16 kg).

**Table 2:** Polymer composition analysis, in terms of orientation of precursor films and membrane crystallinity, membrane pore morphology and membrane permeability.

Sample	*F_c_*	*X_m_ (%)*	Pore Density (pores/μm^2^)	Porous Area (%)	Average Pore Size (µm)	Porosity (%)	Gurley Permeability [µm/(Pa·s)]
**H1**	0.61	57.0	8.7	6.3	0.15	1.01	0.19
**H2**	0.44	63.7	2.1	1.3	0.13	0.23	0.01
**H1-2BR**	0.51	60.9	10.7	6.2	0.13	0.69	0.15
**H1-2VF**	0.57	60.6	6.0	4.7	0.17	0.61	0.14
**H1-10VF**	0.51	60.9	3.7	2.3	0.14	0.47	0.02
**H1-C5**	0.49	57.4	13.6	15.3	0.10	1.81	0.64
**H1-C10**	0.54	58.6	15.5	14.2	0.09	1.75	0.49

**Table 3:** Thermal stability and dynamic-mechanical analysis. T_0.1_ is the temperature at which there was a loss of mass of 10 wt %; T_0.5_ is the temperature at which there was a loss of mass of 50 wt %; T_max_ is the temperature of maximum lost mass velocity.

Membrane	T_0.1_ (°C)	T_0.5_ (°C)	Lost Mass 400 °C (%)	Lost Mass 600 °C (%)	T_max_ (°C)	E’ −30 °C (MPa)	E’23 °C (MPa)	E’ 100 °C (MPa)	E’’ Peak (°C)	Tan δ (°C)
**H1**	300	358	93.0	100	381	8811	2759	828	−0.03	0.12
**H2**	303	370	83.5	100	387	6977	2167	580	−5.74	0.10
**H1-2BR**	301	359	93.0	100	381	6956	1995	681	−0.48	0.11
**H1-2VF**	295	361	92.6	100	380	6921	1994	748	0.56	0.11
**H1-10VF**	292	368	85.3	100	383	6207	1334	662	−3.74	0.10
**H1-C5**	310	377	74.6	94.9	410	10830	3898	1210	−3.14	0.11
**H1-C10**	317	388	63.7	91.7	406	12835	4705	1401	−0.99	0.10

**Table 4:** Draw ratio analysis in terms of orientation, crystallinity, pore morphology and permeability. Gap die, 1.9 mm; cold stage, 0.3 mm/min, 20%; hot stage, 0.3 mm/min, 130%.

Sample	Draw Ratio	*F_c_*	*X_m_(%)*	Pore Density (pores/μm^2^)	Porous Area (%)	Average Pore Size (µm)	Porosity (%)	Gurley Permeability [µm/(Pa·s)]
H1-2BR	**80**	0.51	49.6	12.1	9.8	0.17	0.93	0.18
	**95**	0.54	60.6	12.3	5.9	0.13	0.75	0.16
	**105**	0.55	61.9	13.4	7.7	0.14	0.71	0.16
H1-C10	**60**	0.40	45.2	11.9	12.1	0.09	0.59	0.06
	**70**	0.52	55.5	10.3	11.7	0.09	0.70	0.15
	**80**	0.54	62.9	14.2	14.8	0.10	1.42	0.49
	**90**	0.59	63.5	7.3	11.0	0.11	1.51	0.52
	**100**	0.60	63.9	7.9	10.5	0.11	1.61	0.58

**Table 5:** Uniaxial strain analysis in terms of orientation, crystallinity, pore morphology and permeability. Gap die 1.9 mm; draw ratio 90.

Cold Stage		Hot Stage		*X_m_(%)*	Pore Density (pores/μm^2^)	Porous Area (%)	Pore Size (µm)	Porosity (%)	Gurley Permeability [µm/(Pa·s)]
Strain Rate (mm/min)	Extent (%)	Strain Rate (mm/min)	Extent (%)						
0.3	35	0.3	320	62.1	9.3	20.2	0.36	1.28	0.29
1.0		1.0		62.5	10.5	17.0	0.25	1.05	0.24
0.3	35	0.3	320	62.1	9.3	20.2	0.36	1.28	0.29
50.0				62.2	8.9	18.1	0.33	1.22	0.25
0.3	20	0.3	130	60.7	9.4	14.6	0.25	1.21	0.22
		50.0		59.2	12.9	4.9	0.09	0.51	0.07
1.0	20	1.0	320	57.4	6.8	13.9	0.32	0.97	0.22
	35			62.5	10.5	17.0	0.25	1.05	0.24
	55			58.9	8.9	11.3	0.21	0.95	0.20
0.3	20	0.3	65	60.8	9.3	2.8	0.09	0.30	0.02
			100	59.2	14.0	5.4	0.11	0.55	0.08
			130	60.7	9.4	14.6	0.25	1.21	0.22
			300	65.6	7.8	18.8	0.35	1.25	0.26
